# Based on improved deep convolutional neural network model pneumonia image classification

**DOI:** 10.1371/journal.pone.0258804

**Published:** 2021-11-04

**Authors:** Lingzhi Kong, Jinyong Cheng

**Affiliations:** School of Computer Science and Technology, Qilu University of Technology (Shandong Academy of Sciences), Jinan, China; Politechnika Slaska, POLAND

## Abstract

Pneumonia remains the leading infectious cause of death in children under the age of five, killing about 700,000 children each year and affecting 7% of the world’s population. X-ray images of lung become the key to the diagnosis of this disease, skilled doctors in the diagnosis of a certain degree of subjectivity, if the use of computer-aided medical diagnosis to automatically detect lung abnormalities, will improve the accuracy of diagnosis. This research aims to introduce a deep learning technology based on the combination of Xception neural network and long-term short-term memory (LSTM), which can realize automatic diagnosis of patients with pneumonia in X-ray images. First, the model uses the Xception network to extract the deep features of the data, passes the extracted features to the LSTM, and then the LSTM detects the extracted features, and finally selects the most needed features. Secondly, in the training set samples, the traditional cross-entropy loss cannot more balance the mismatch between categories. Therefore, this research combines Pearson’s feature selection ideas, fusion of the correlation between the two loss functions, and optimizes the problem. The experimental results show that the accuracy rate of this paper is 96%, the receiver operator characteristic curve accuracy rate is 99%, the precision rate is 98%, the recall rate is 91%, and the F1 score accuracy rate is 94%. Compared with the existing technical methods, the research has achieved expected results on the currently available datasets. And assist doctors to provide higher reliability in the classification task of childhood pneumonia.

## Introduction

Pneumonia refers to the inflammation of the bronchioles, bronchi, alveoli, and interstitial lungs in the lungs. It is mostly caused by pathogenic microbial infections, stimulation of physical and chemical factors, immune function damage, as well as allergies and drug factors. Among them, bacterial and viral pneumonia are the most common pneumonia, which poses a great threat to the health of children and the elderly. In recent years, despite the use of powerful antibiotics and effective vaccines, the overall mortality rate of pneumonia has increased. Therefore, pneumonia is still a disease that requires active treatment and prevention [1]. The World Health Organization (WHO, Geneva, CH) has also stated that one of the highest causes of deaths of children under 5 years of age today is pneumonia, which causes approximately 1.4 million deaths, accounting for approximately 18% of the world’s children under 5 years of age [2]. The top five causes of death in low-income countries is pneumonia. Proficiency in the use of computer-aided diagnosis (CAD) technology is likely to help doctors and patients detect different types of abnormalities in medical images, so that they can analyze and diagnose diseases more accurately and in a timely manner [3–5].

**Fig 1:**
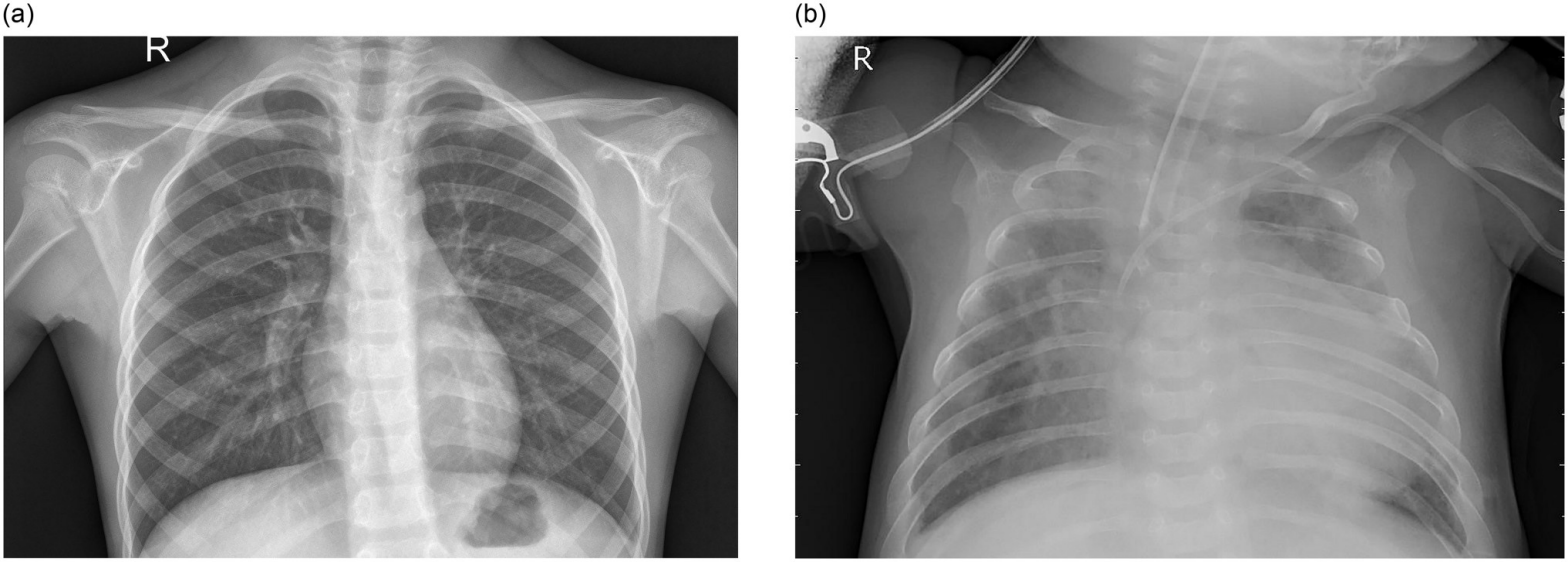
(a): Normal chest X-ray image, (b): Chest X-ray image of patients with pneumonia.

There are many examination methods for lung diseases, such as chest X-ray, chest X-ray, chest CT, magnetic resonance imaging (MRI), lung function, bronchoscopy, etc., which are commonly used examination methods, and are common examples of CAD programs that help diagnose lung diseases [6–8]. Although there are many options for detecting lung diseases, for most developing countries, medical resources are still scarce. In comparison, the accuracy of chest X-ray images (CXRAY) cannot be completely equal to the diagnosis of chest CT and MRI. However, the use of CXRAY is still the most needed radiological examination [3]. Fig 1 [33] shows the lungs of normal people and patients with pneumonia under X-ray irradiation.

Although, sometimes the appearance of pneumonia in CXRAY is often vague and may be confused with other diseases. These inaccuracies have led to a wide range of biased decisions and diversity among radiologists in diagnosing pneumonia. However, the current CXRAY technology is also constantly being improved. If it is paired with a skilled and well-trained doctor, the accuracy of pneumonia detection will also be greatly improved [9].

Nowadays, in order to further promote and speed up childhood pneumonia, deep learning is an important branch in the field of artificial intelligence. It can use raw data as the input of the algorithm, through the input data, feature extraction, stratified sampling, and raw data layer by layer Abstract the target characteristics required by one’s own task. Finally, the learned characteristics are mapped to the task goal as the end, and the whole process does not require manual operation. For example: In the literature [10], it is proposed to use the support vector machine method to identify the period of the lung nodule, so as to judge whether it is benign or malignant. In the literature [11], the use of AlexNet technology to identify pneumonia in X-ray films combined with support vector machine technology and Softmax greatly improves the accuracy. The development of deep learning is inseparable from the study of the principles of brain cognition. Deep learning is a branch of artificial neural networks (artificial neural networks). Now, a neural network with a deep network structure is the earliest network model in deep learning. For radiologists, X-ray image analysis and inspection are an indispensable part of their daily work. The work is cumbersome and critical, and it also consumes a lot of time and energy. Therefore, researchers have proposed several computer algorithms to analyze X-ray images by combining with computers [12]. Machine learning (ML) technology is used in various fields of medicine, and has excellent results in the detection of pneumonia, breast cancer, heart disease, cataract and other diseases. The combination of ML technology and medical treatment can provide convenience to more people with minimal cost, especially for countries such as India, South Asia and Sub-Saharan Africa.

In this research, we proposed a method that uses Transfer learning [13, 14] technology as the initial data, using Xception algorithm [15] and long short-term memory algorithm (LSTM) [16] to merge, from X-ray images of children’s lungs can distinguish whether they are infected with pneumonia. The results of the model are compared and analyzed comprehensively with other studies in the literature. The Xception-LSTM model used in this experiment can better assist doctors in the diagnosis and treatment of patients with pneumonia, and can detect children in a timely and effective manner. Can better get timely treatment. Even if this model cannot completely replace manual classification, it can still reduce the burden of doctors’ detection, improve safety, reliability, and more accurately classify pneumonia.

## Related work

The in-depth research of CNN algorithm has been widely used in various fields, such as industry, medicine, edge computing, flow control, etc. Some studies using convolutional neural networks have also achieved early results in medicine. Use convolutional neural networks in the detection of diabetic retinopathy [17]. Nowadays, many researchers also regard lung sounds as an important measure to diagnose lung diseases. A single entropy nullification classification is less accurate for lung sound, compared with seven entropies combined can be as high as 94.95% [18]. Severe smokers can accept low-dose chest examination. CT can automatically detect coronary artery, thoracic aorta and heart valve calcification. The cardiovascular risk assessment can be reliably achieved [19]. It combines supervised learning back propagation neural network and unsupervised learning competitive neural network to diagnose pneumonia diseases [20]. Lakhana and Sundaram scholars use neural network and AlexNet technology to partition and enhance the data, without any pre-training to obtain 0.94–0.95 under the curve [21]. In medical imaging, the research established a special diagnostic tool based on a deep learning framework, which can better screen patients with common treatable blind retinal diseases [22]. In the literature [23], Santosh and Antani once proposed the use of front chest radiographs to screen AIDS-positive patients for tuberculosis. When diagnosing pneumonia, chest X-ray is also the most indispensable step. In the literature [24], using the VGG16 model in the case of unclear images to diagnose pneumonia, an accuracy rate of 90.54%, 98.71% recall rate and 87.69% accuracy (comparison). Husanbir Singh Pannu et al. [25], once established a reliable neuro-fuzzy inference system (ANFIS) and particle swarm optimization (PSO) to improve the classification rate. Shawni Dutta et al. [26] used a combined model composed of long-term short-term memory (LSTM) and gated recursive unit (GRU), and incorporated memory cells into neural networks for training and testing. Literature [27] once proposed the use of hybrid neuro-heuristic approach and image descriptors of the spatial distribution of chromaticity, saturation, and brightness values to accurately detect diseased and degraded tissues in lung X-ray images. The moth-Flame algorithm is more effective for pneumonia detection. In the literature [28], the fitness function and search model of Bio-Inspired Methods are used as decision support for the detection of lung lesions, and can better guide doctors to find areas that may have lesions. In [29], a fuzzy logic segmentation method and on a probabilistic neural network (PNN) are applied to form an evaluation model for lung cancer. The fuzzy set is used as a segmentation tool to send the results to the neural network for evaluation. As a variant structure of recurrent neural net-work (RNN), LSTM can better mine sequence time information As a variant structure of recurrent neural net-work (RNN), LSTM can better mine sequence time information [30–32] and realize image classification. and realize image classification.

In general, deep learning methods have made considerable achievements in medical images. This research also further innovated and improved on this basis. The third part focuses on data preprocessing to enhance the readability and clarity of data. The fourth part is to combine the new model based on deep learning and transfer learning based on LSTM technology to optimize the accuracy of the model and improve the optimization ability. In order to strengthen the relevance between categories, the idea of feature selection is appropriately improved. The ultimate goal of this experiment, as well as the main motivation for the development of this work, is to use artificial intelligence to better automate the classification of diseases in the medical field.

## Dataset and preprocessing

### Dataset

The dataset mainly used in this study was published by Kermuny et al. in 2018 [33]. This dataset has 5863 x-ray images (JPEG) and two categories (normal images/images with pneumonia), which are derived from children aged 1 to 5 years old from the Guangzhou Women and Children’s Medical Center. All chest x-ray imaging [33] is part of the routine clinical care of the patient. In order to improve the analysis and evaluation of the accuracy of chest radiographs, a total of 5856 chest X-ray images were collected and marked by screening the quality. Taking into account comprehensively, due to external factors, such as the scanning location, personal habits, and the patient’s experience of other diseases, the image is unclear. In the research process, we will enhance the image to improve readability.

### Data image preprocessing

In medical images, we usually hope that the brightness of the image is evenly distributed. However, the brightness changes are random, which reduces the image quality and produces visual noise, which is also easy to cause troubles for the doctor during diagnosis [34, 35]. Therefore, before the experiment, we performed the image denoising processing. Since some areas in the lung X-ray image look very similar, in order to reduce the error of diagnosis, the non-local mean denoising algorithm was selected in the experiment. Take a small window around it, scan the image to obtain similar windows, average all the windows, and then calculate the result to replace the pixel [36].

We store the noise-reduced image in a new folder. In order to ensure the quality of the image, we artificially enhance the image through computer means. Data enhancement has basically become the simplest and direct way to improve model performance. Taking appropriate measures to increase the image quality will also help to solve the over-fitting problem, and better improve the generalization ability of the image in the training process, making the application range more extensive and more accurate [37]. For example, Table 1.

**Table 1:** Enhanced processing methods for data.

Data enhancement processing	size
Scaling	1/255.0
Reflection Transformation	7
Width range of movement	0.5
Altitude range of movement	0.45
Shear range	0.2
Zoom range	0.45
Flip the image horizontally	True

In the training set, we scale and increase the transformation of the image. The study set a zoom factor of 1/255. The purpose of zooming is to enlarge or reduce the length and width of the image. The overall size of the changed image is not to cut the image. The rotation range represents the angle at which the image is randomly rotated (rotated around the center of the image) during training, which is 7 degrees. The height displacement represents a 0.2% movement of the image in the vertical direction. The width displacement is the same as the height displacement, except that the angle changes from the vertical direction to a 0.2% change in the horizontal direction. In the above process of enhancing the image, the size will be randomly enlarged or reduced by 0.45 times to enhance the image, and finally the image needs to be flipped in the horizontal direction again [37].

## Model

### Transfer learning

Transfer learning, as the name suggests, is to transfer the parameters of the trained model (pre-training model) to the new model to help the new model training. In the medical field, we often need a large number of images for analysis and diagnosis, training a deep CNN [38] model, such as ResNet, Xception, or Vgg, but also a large number of datasets, because they contain millions of trainable Parameters, a small dataset will not be enough to get a good generalization of the model. First, learn about the application of the model by learning the Xception model, and at the same time combine other model structures, such as wavelet transform, residual network, Long-term and short-term memory artificial neural network (LSTM), etc. This article combines Xception and LSTM models to perform with other CNN structures Compared. Depthwise Separable Convolution was first proposed by Dr. Laurent Sifre in literature [39].

The Google team proposed in 2014 that GoogLeNet has improved the traditional convolutional layer. The so-called Inception structure is that different sizes of convolution kernels can be used in the same layer. This innovation has made CNN a small breakthrough. Subsequently, improved versions of Inception V2 [40], Inception V3 [41], and Inception V4 [42] have been proposed. First of all, in Inception V2, the traditional structure often uses the form of a 5 × 5 large convolution kernel. However, in the V2 structure, two 3 × 3 convolutions are used to increase the depth of the network and to a certain extent. Network effect, the establishment of more complex nonlinear transformation, on the other hand, in order to reduce the difficulty of neural network training, choose to use Batch Normalization to achieve, for example, Eq (1).

$$\begin{array}{c} f{\left(x\right)}^{\left(t\right)}=\alpha^{\left(t\right)}\hat{x}+\beta^{\left(t\right)} \end{array}$$ (1)

*f*(*x*) and *β* are expressed as parameters; $\hat{x}$ is the normalization term of a batch.

**Fig 2:**
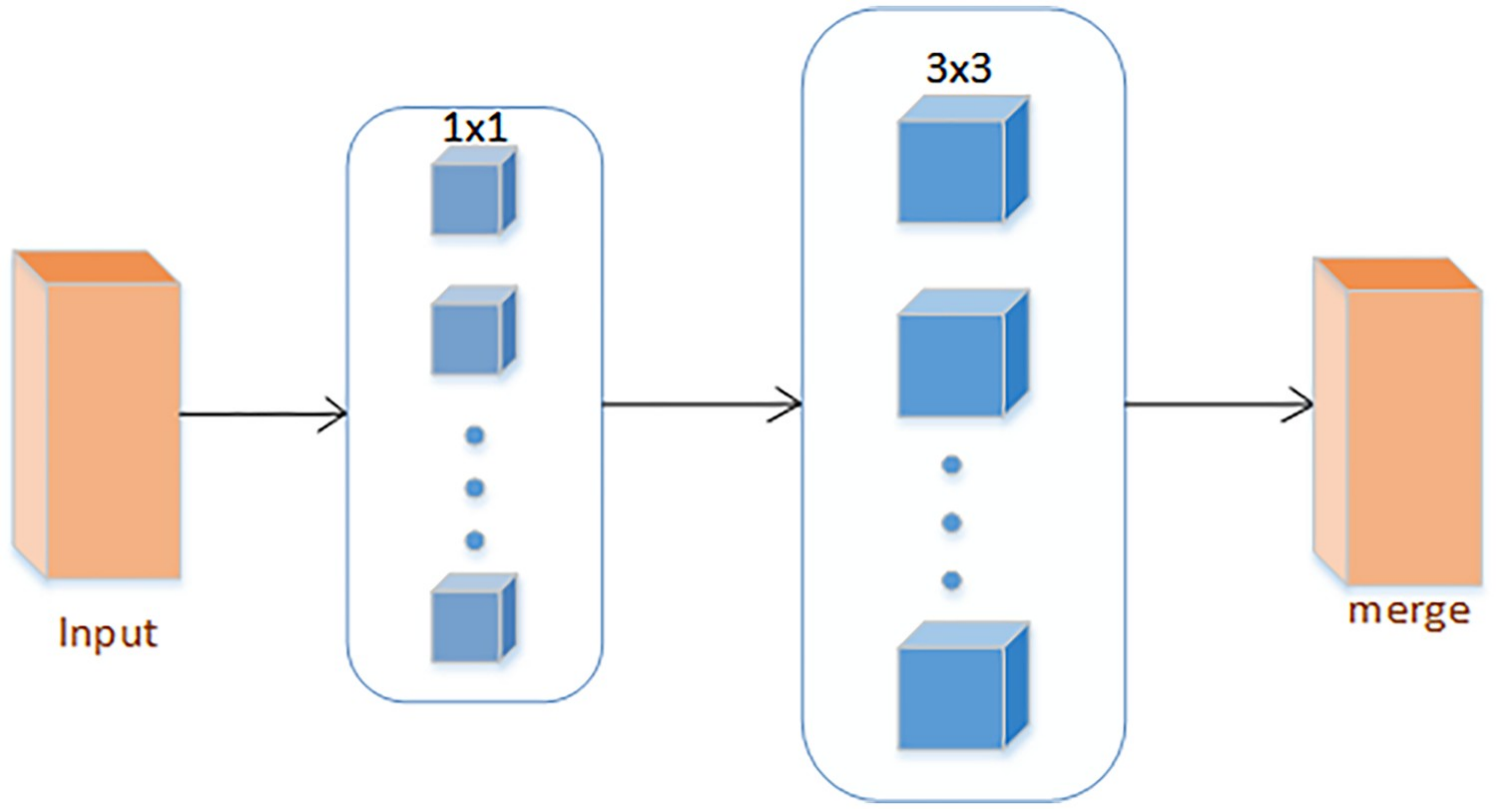
Simplified inception structure diagram.

**Fig 3:**
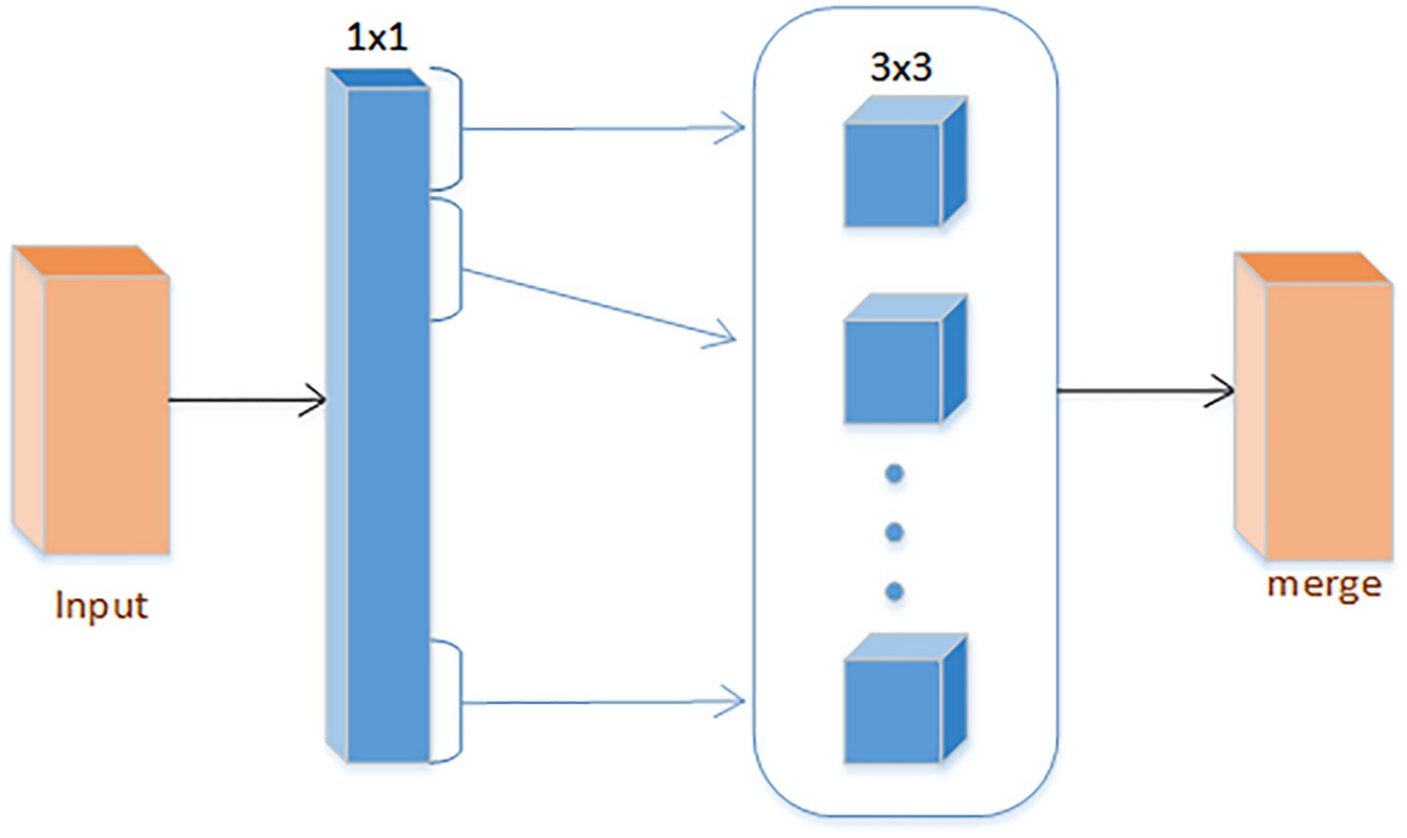
Enhanced inception structure.

Inception V3 has more types in the Inception Module, combined with the use of branching technology [43], and then split a larger two-dimensional convolution into two smaller one-dimensional convolutions, and at the same time, the standard Inception Simplify, as shown in Fig 2, choose to use only one form of convolution (for example: 3 × 3 convolution kernel), remove the average pooling structure, reduce a large number of parameters to accelerate calculations and reduce over-fitting; at the same time add a layer Non-linear transformation expands the expressive ability of the model. Xception is also an improvement based on Inception V3, using deep separable convolution. An “extreme” version of the initial module, shown in Fig 3, first uses 1 × 1 convolution to draw cross-channel correlation, and then independently draws the spatial correlation of each output channel [44].

The Xception network in this experiment mainly uses separable convolution, which replaces the convolution operation in Inception V3. At the same time, it can further improve the effect of the model without increasing the complexity of the network model, as shown in Table 2.

**Table 2:** The number of parameters of xception-CNN.

Layer	Type	Details
1	Conv2D	filters = 32,kernel_size = (3,3),strides = (2, 2),activation = ‘relu’
2	Conv2D	filters = 64,kernel_size = (3,3),activation = ‘relu’
3	SeparableConv2D	filters = 128,kernel_size = (3,3),activation = ‘relu’
4	SeparableConv2D	filters = 128,kernel_size = (3,3),activation = ‘relu’
5	MaxPooling2D	kernel_size = (3,3),strides = (2, 2)
6	SeparableConv2D	filters = 256,kernel_size = (3,3),activation = ‘relu’
7	SeparableConv2D	filters = 256,kernel_size = (3,3),activation = ‘relu’
8	MaxPooling2D	kernel_size = (3,3),strides = (2, 2)
9	SeparableConv2D	filters = 728,kernel_size = (3,3),activation = ‘relu’
10	SeparableConv2D	filters = 728,kernel_size = (3,3),activation = ‘relu’
11	MaxPooling2D	kernel_size = (3,3),strides = (2, 2)
12	SeparableConv2D	filters = 728,kernel_size = (3,3),activation = ‘relu’
13	SeparableConv2D	filters = 728,kernel_size = (3,3),activation = ‘relu’
14	SeparableConv2D	filters = 728,kernel_size = (3,3),activation = ‘relu’
15	SeparableConv2D	filters = 728,kernel_size = (3,3),activation = ‘relu’
16	SeparableConv2D	filters = 1024,kernel_size = (3,3),activation = ‘relu’
17	MaxPooling2D	kernel_size = (3,3),strides = (2, 2)
18	SeparableConv2D	filters = 1536,kernel_size = (3,3),activation = ‘relu’
19	SeparableConv2D	filters = 2048,kernel_size = (3,3),activation = ‘relu’

### Xception+LSTM

**Fig 4:**
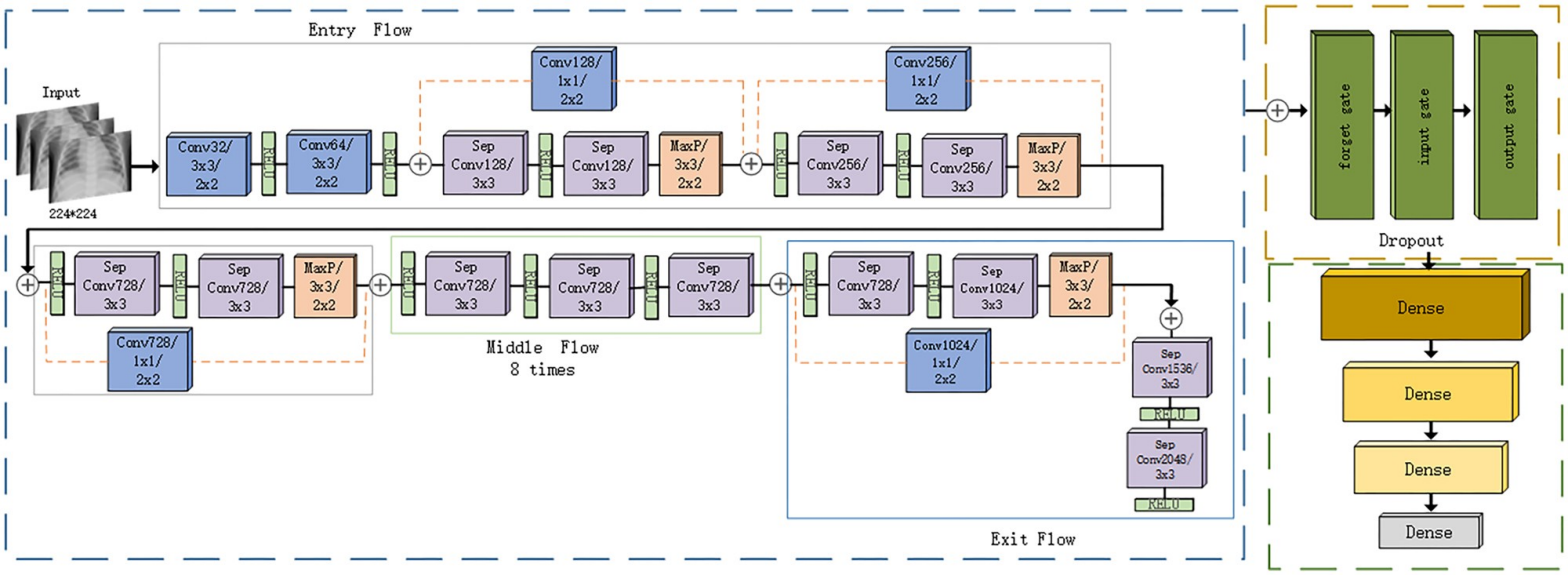
Fusion of Xception and LSTM models.

Convolutional neural network (CNN) in image recognition is a multi-layer neural network composed of convolutional layers for feature extraction and sub-sampling layers for feature processing, and has an indispensable position in the field of computer vision [45]. This study adopts the Xception backbone model based on CNN, which can effectively obtain the data of pneumonia image characteristics. But they lack certain performance in the learning dependence of cross-sequence information, and cannot handle sequential data. However, LSTM is a special recurrent neural network that can learn the long-term dependence of data while retaining information in the form of sequences. Therefore, in order to better extract pneumonia image features in the experiment, we propose a model combining Xception network and long short-term memory artificial neural network (LSTM) in this article, as shown in Fig 4. Then through the training image dataset, the network’s batch_size, image quality enhancement and other parameters are fine-tuned to improve the model accuracy and generalization ability. Finally, the neural network attention mechanism is used to merge and analyze the features generated by the two models of Xception and LSTM, and the features are normalized and serially fused into fusion features through the fully connected layer FC [46].

The Xception model feature extraction cross-channel correlation and spatial correlation mapping can be more integrated. At the same time, the architecture has 36 convolutional layers, which can be divided into 14 modules, and the overall will be divided into input blocks (Entry Flow), Intermediate block, output block. Except for the first and last modules, all of these modules have linear residual connections, which are completely based on the convolutional neural network architecture of the deeply separable convolutional layer [15], which forms the basis of the feature extraction of the network. In the first part of the Xception network, an input image of 224 × 224 × 3 is obtained and sent to the input block. After an intermediate block of eight iterations, it is finally output by the input block. Output four-dimensional pneumonia feature data, and convert the data into three-dimensional data that can be read by the LSTM layer and send it to the second half of the model architecture. There are 200 neural units in an LSTM layer that go through three stages, namely forgetting Stage, select memory stage, output stage. The output data is fully connected through four layers of dense, and the number of neurons in the first three layers decreases at a rate of 2 times decreasing layer by layer. At the same time, with the RELU activation function, the previous local features are reassembled into a complete graph through the weight matrix. In the last layer, predictive classification is performed by selecting the sigmoid activation function.

In the LSTM layer, the unimportant input features are selected and forgotten. Among them, the passed *p*^*m*^ changes very slowly. Usually the output *p*^*m*^ is *p*^*m*−1^ passed from the previous state plus some values, as shown in Eq (2). Among them, *p*^*m*^, *n*^*m*^, and *g*^*m*^ are multiplied by the splicing vector by the weight matrix *W*, and then converted into a value between 0 and 1 through a “sigmoid” activation function, which is used as a gated state. As in Eq (3), *c* is to convert the result into a value between -1 and 1 through a “tanh” activation function. Therefore, use the gating state, such as Eq (4), to control the transmission state, remember the long-term memory, and forget the unimportant information.

$$\begin{array}{c} p^{m}=c^{r}⊗p^{m-1}+c^{s}⊗c \end{array}$$ (2)

$$\begin{array}{c} n^{m}=c^{t}⊗\text{tanh}(c^{m}) \end{array}$$ (3)

$$\begin{array}{c} g^{m}=\delta (W_{i}n^{m}) \end{array}$$ (4)

### Optimizing the loss function

In this experiment, in order to better improve the classification results, there are different degrees of optimization and improvement in the optimizer and loss function. As we can see from Table 3, due to the poor ability of other optimizers to extract features under the same dataset, the RMSprop (root mean square) optimizer is selected in this experiment. The purpose is to make the data better approximate or reach the optimal value, and speed up The gradient descent algorithm adjusts the step size when updating the parameters. The step will be smaller in the direction of drastic change, and the step will be larger in the direction of gentle change. For gradient descent, RMSprop moves in the direction of the horizontal axis, and there will be large fluctuations in the vertical axis. RMSprop can automatically adjust the parameters through the following Eqs (5) and (6), so that the gradient difference between variables can be effectively alleviated.

$$\begin{array}{c} T_{dv}=\alpha T_{dv}+\left(1-\alpha \right){\left(dv\right)}^{2} \end{array}$$ (5)

$$\begin{array}{c} T_{db}=\alpha T_{db}+\left(1-\alpha \right){\left(db\right)}^{2} \end{array}$$ (6)

*dv*^2^ and *db*^2^ represent the calculation of the square of each component of the vector. The expansion of *T*_*dv*_ and *T*_*db*_ is also a vector, and each component is an exponentially weighted moving average of the square of the corresponding component values of weights dv and db, and controls the weighted average.

$$\begin{array}{c} V=V÷\gamma \frac{dv}{\sqrt{T_{dv}}} \end{array}$$ (7)

$$\begin{array}{c} b=b÷\gamma \frac{db}{\sqrt{T_{db}}} \end{array}$$ (8)

*v* represents the parameter on the horizontal axis, and its rate of change is very small, so the value of *dv* is very small, so T_dv_ is also small, and *b* fluctuates greatly on the vertical axis, so the slope is particularly large in the *b* direction. Therefore, among these differentials, *db* is larger and *dv* is smaller. In order to balance the range of changes between T_dv_ and T_db_, the parameter values *V* and *b* are updated. The divisor of *V* is a small number. Generally speaking, the change of *V* is large, and the change of *V* can be expressed by Eq (7). The divisor of *b* is a larger number, so the update of *b* in Eq (8) will be slowed down, and the vertical change will be relatively smooth. The two hyperparameters *γ* are used to slow down the swing when the parameters are falling, and allow you to use a larger learning rate to speed up the algorithm.

**Table 3:** Test optimizer.

Structure	Size	Iteration	Optimizer	Learning rate
Xception+LSTM	224×224	80	RMSprop	0.0001
Xception+LSTM	224×224	80	Radam	0.0001
Xception+LSTM	224×224	80	SGD	0.0001
Xception+LSTM	224×224	80	Adam	0.0001

In the logistic regression binary classification problem, we usually use sigmoid to map the output to the [0, 1] interval to classify with 0.5 as the limit, and the sigmoid function is generated by the principle of maximum entropy. In this experiment, when binary_crossentropy is used, the error can be compared. When it is large, the weight update speed is increased; when it is small, the weight update is slow, but the traditional cross-entropy also has certain problems, although there are significant features in terms of features. Advantages, but the similarity between the relevant classes within the vector is still relatively lacking. We can use the chi-square test to compare two or more samples (composition ratio) and the correlation analysis of two categorical variables. At the same time, it is possible to calculate the degree of agreement between actual and predicted frequencies or the problem of goodness of fit.

We can assume that the output probability of the data predicted by the model is $\hat{p}=[{\hat{p}}_{1},{\hat{p}}_{2},\ldots ,{\hat{p}}_{t}]$, and the probability of the true label value is *p* = [*p*_1_, *p*_2_, …, *p*_*t*_], and we use *t* to represent the total amount of data. From this, this research can propose a method based on binary_crossentropy, which incorporates the feature selection method proposed by Pearson (as shown in Eq (9)), which can be expressed as Eq (10):

$$\begin{array}{c} x^{2}=\frac{{\left(p-\hat{p}\right)}^{2}}{\hat{p}} \end{array}$$ (9)

$$\begin{array}{c} L=-\frac{1}{m}\sum_{j=1}^{m}[p\text{log}\left(\hat{p}\right)+\left(1-p\right)\text{log}\left(1-\hat{p}\right)+\frac{{\left(p-\hat{p}\right)}^{2}}{\hat{p}}] \end{array}$$ (10)

*x*^2^ represents the degree of difference between the actual value and the predicted value, *p* represents the current true label value, $\hat{p}$ represents the current predicted label value, and *L* represents the loss function of this experiment.

### Number of balanced categories

**Fig 5:**
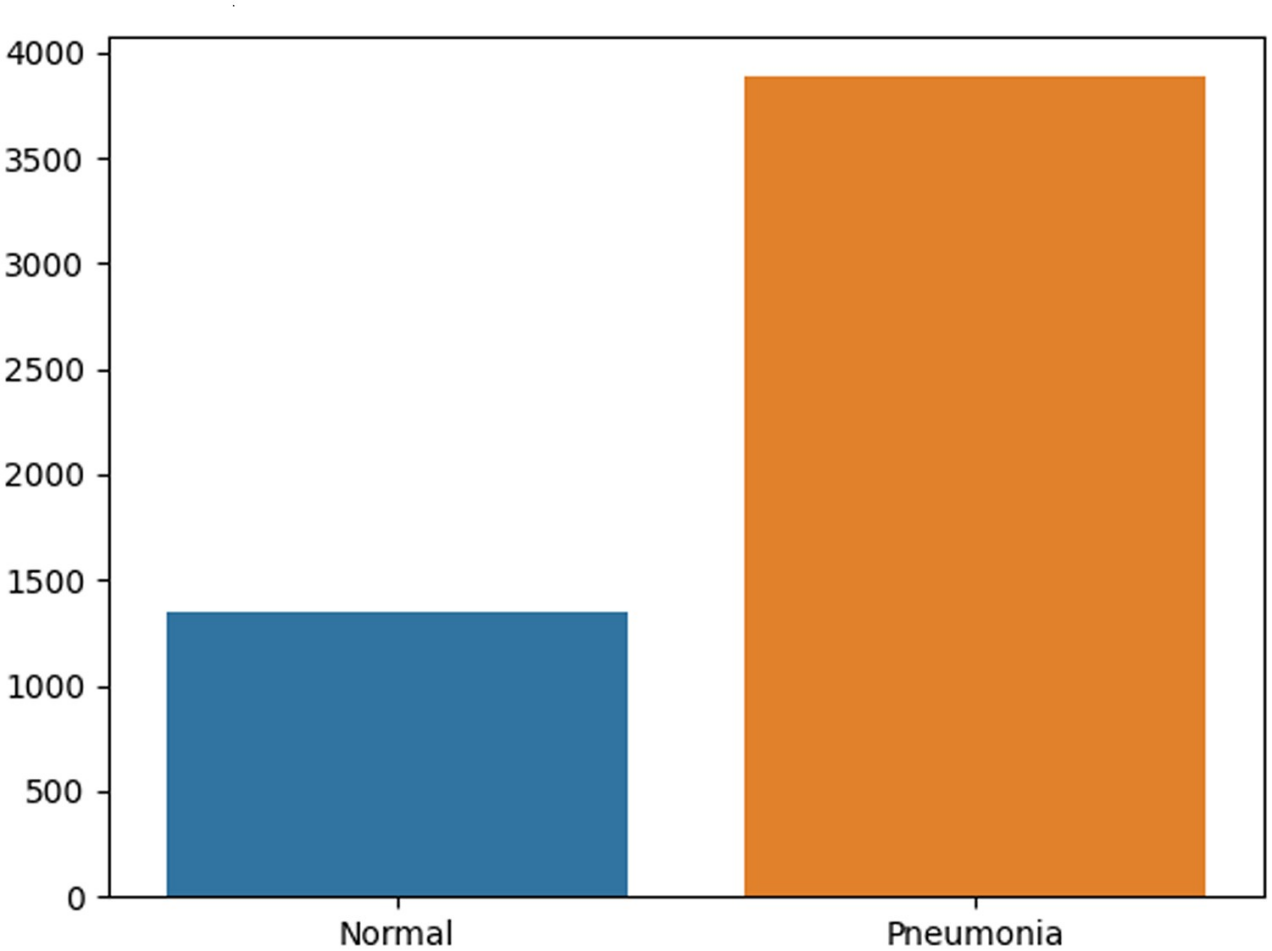
The number of normal and pneumonia images in the training set.

By dividing the dataset, it is found that the number of patients with pneumonia is significantly higher than the normal number of patients, and the sample is not balanced, resulting in the sample is not an unbiased estimate of the overall sample, which may lead to a decline in the predictive ability of our model. If the minority class is given a very high class weight, the algorithm is likely to be biased towards the minority class and will increase the error in the majority class. In this case, we can try to solve this problem by adjusting the sample weight. In order to make an accurate judgment on the imbalance between the two, first of all, through Fig 5, it is obvious that the category imbalance in the training set can be seen visually. For further verification, it can be seen from [3] that the use of computational training set Whether the imbalance ratio *IR* is greater than 1.5, to judge whether the training set is balanced.

$$\begin{array}{c} IR=\frac{|\;\text{Max\_class}|}{|\;\text{Min\_class}|} \end{array}$$ (11)

$$\begin{array}{c} IR=\frac{|\;\text{Pneumonia}\;|}{|\;\text{Normal}\;|}=\frac{3883}{1349}\approx 2.88 \end{array}$$ (12)

It can be seen from the requirements that the *IR* does not meet the range of < 1.5, and the data between the two categories in the training set is not balanced. The imbalance of the amount of data will cause the classification performance to change. Therefore, in order to balance the category ratio more reasonably, the weight value is selected to balance and the hyperparameter category weight is modified. By default, the value of class_weights is “None”, that is, the weights of the two classes are equal. When the classification situation of our training set is somewhat uneven, we can change None to “balanced”, which can better optimize the weight ratio of the two. The formula for calculating the weight under “balanced” can be expressed as:

$$\begin{array}{c} W_{m}=i_{-}\text{sum}/(i_{-}\text{class}\;\times i_{{\text{sum}}_{m}}) \end{array}$$ (13)

- *W*_*m*_: Represents the final calculated weight value of each class.
- *i*_sum: indicates the size of all samples in this type of dataset.
- *i*_class:indicates the number of categories in the total sample.
- *i*_*Sum*_*m*_: indicates the number of samples corresponding to the m category.

The weight values of normal persons and pneumonia patients in the training set under the condition of “balanced” are calculated by Eq (13), respectively, *W*_1_ = 1.94(N), *W*_2_ = 0.68(P), when the class weight = “balanced”, the model will Automatically assign class weights that are inversely proportional to their respective frequencies. Purposely increase the power of the minority class and reduce the power of the majority class, thereby optimizing the imbalance between categories.

### Ingredient-sensitive learning

Classification is an indispensable part of the machine learning process. However, different sample differences can easily affect the classification results. In this experiment, in order to better balance the dataset, we first processed and enhanced the dataset (through rotation, translation, zooming, etc.) so as to reduce the data loss rate to a certain extent. Secondly, when processing the data in this experiment, the samples are balanced by modifying the hyper-parameter category weight “balanced”. Through the adjustment of the hyper-parameters, the greater the number of categories, the smaller the penalty item, and the more input samples of a certain category. The smaller the penalty term of the first category is, the learning bias problem caused by the imbalance of the input samples can be well balanced. Finally, on the basis of binary_crossentropy loss, the basic idea is to check the degree of fit, and to further integrate the correlation between the two loss functions. Improve the correlation between actual samples and predicted samples, and enhance learning ability.

## Experimental results

### Confusion matrix, ROC

**Fig 6:**
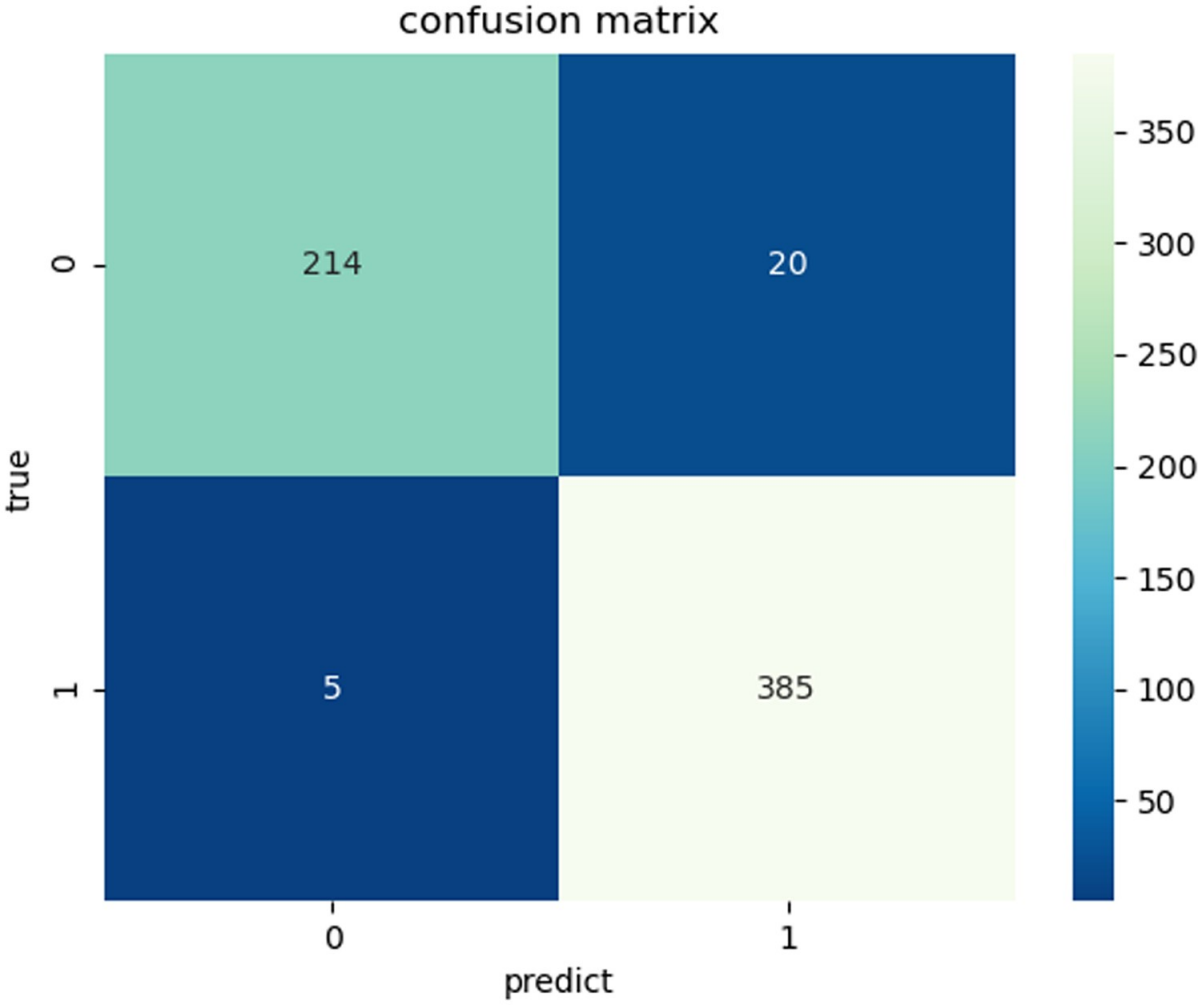
Classification model evaluation index-confusion matrix.

When we are conducting experiments, the more accurate the results of the experiments, the higher the quality of the experiments. We also know the four basic indicators from the above. The confusion matrix is the number of observations that are classified into the wrong class and classified into the right class by the statistical classification model. It can be seen from Fig 6 that through this experimental model, the predicted correct positive examples (TP) and the predicted correct negative examples (TN) account for about 95% of the total number of tests, indicating that most of the data predictions are relatively accurate.

**Fig 7:**
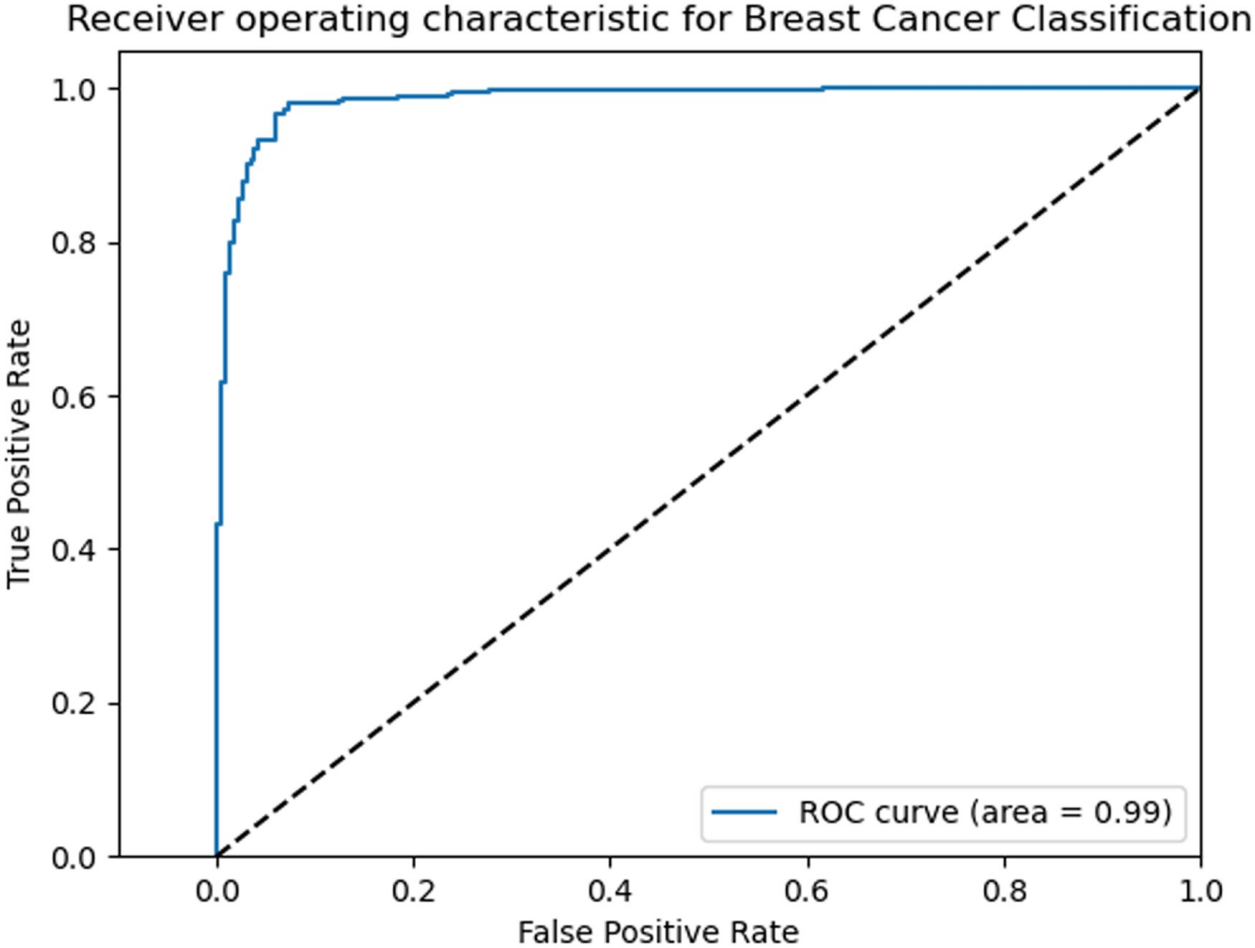
Rate of change indicator.

In this section, we will analyze the confusion matrix and ROC curve in this experiment based on the above-mentioned classification model. ROC is equivalent to a random classifier that visualizes the true class rate and the negative positive class rate, and makes decisions based on the threshold. The larger the FTR value on the horizontal axis, the greater the number of positive samples and the greater the number of negative samples predicted. On the vertical axis TPR, the more positive samples there are, the more positive classes are predicted. Therefore, the overall evaluation of the ROC curve compares the performance of the classifiers tested in experiments. More stable to experimental evaluation. As shown in Fig 7.

AUC (Area under the Curve of ROC) is the area under the ROC curve, which is the standard to judge the merits and demerits of dichotomous prediction model. As can be seen in Fig 7, the value of AUC can reach 99% through the operation of the data in this model, and its value more intuitively reflects the model classification ability expressed to us by the ROC curve. Its value (the larger the better) represents The performance of the model is good or bad.

### LOSS curve, experimental results

**Fig 8:**
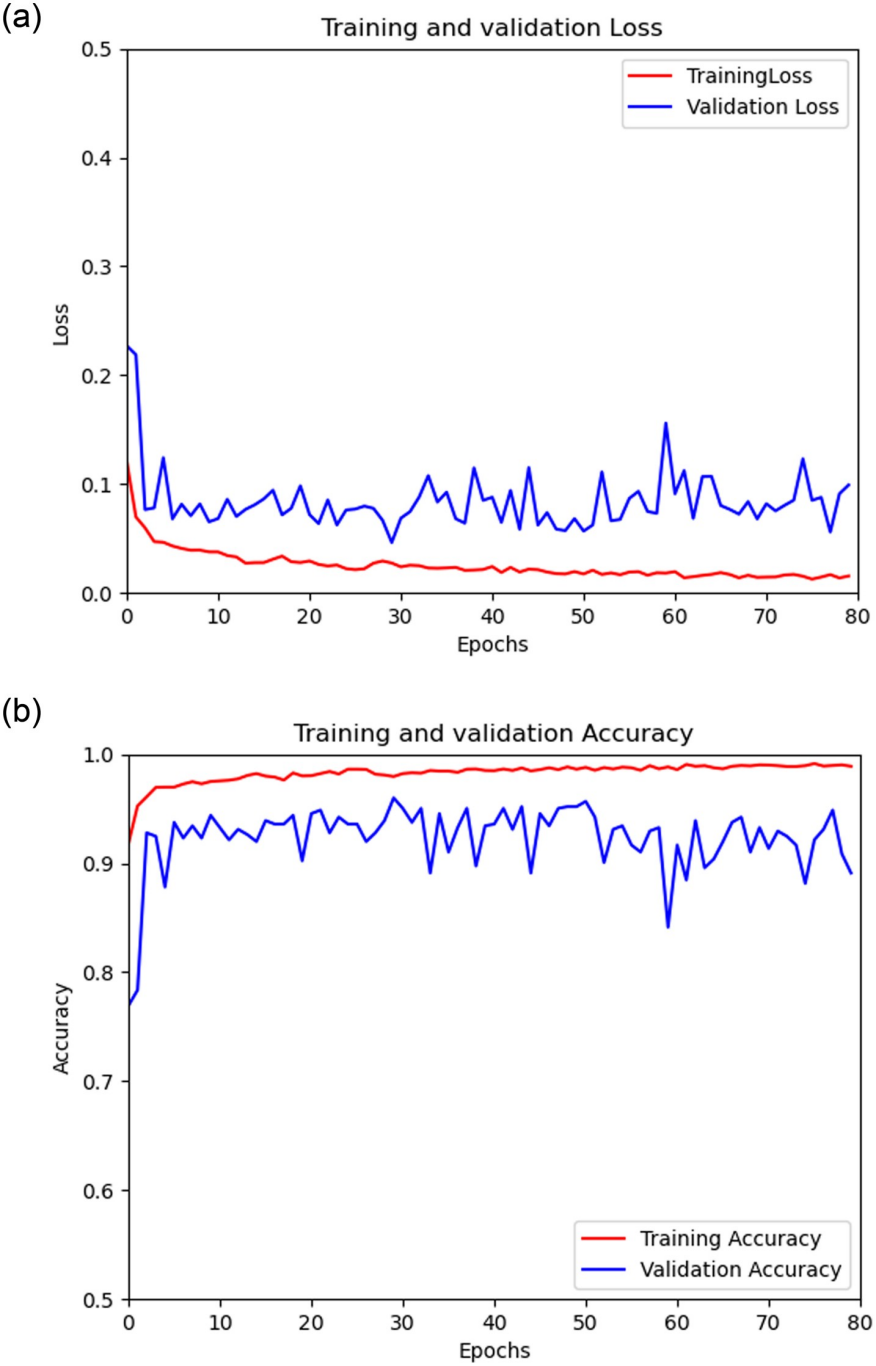
(a): Schematic diagram of loss change, (b): Schematic diagram of accuracy rate change.

Through the connection and fusion between the Xception convolutional neural network model and the LSTM model, compared with the model only using multiple convolutional neural networks for prediction, the accuracy rate can be seen to be significantly improved, as shown in Table 4. At the same time, it is effective to use the RMSprop (root mean square) optimizer and add a loss function based on binary_crossentropy loss combined with interval estimation and hypothesis testing ideas to train the network model. Fig 8 shows the convergence of the training loss in this experiment.

**Table 4:** Comparison of different experimental work data.

Research work	Accuracy rate	Recall rate	Precision rate	F1
Kermany et al.(2018) [22]	0.93	0.93	0.90	-
Stephen et al.(2019) [37]	0.94	-	-	-
Ayan et al.(2019) [47]	0.87	0.91	0.82	0.92
Pant et al.(2020) [48]	0.90	0.87	0.99	0.92
Hu et al.(2020) [49]	0.93	0.97	0.87	-
Our method	0.96	0.91	0.97	0.94

The initial training loss of Loss is about 0.27, and the test loss is about 0.13. During the 5 iterations of the data, the rate of decline was rapid, and then the decline was relatively slow, but the overall decrease. For Accuracy, the accuracy of the first three iterations can be as high as 90% quickly, and the subsequent overall show a slow upward trend. Generally speaking, after Fig 8, we can observe that the accuracy increases with the passage of time, and the overall loss is also decreasing.

It can be seen from Table 4 that this experiment compares the effects of different mainstream models when relying on a large data set.

If the classification of pneumonia detection can be improved more effectively and accurately, the probability of children dying from pneumonia disease can be greatly reduced. Use computer-aided medical diagnosis to automatically detect lung abnormalities and improve the accuracy of doctors’ diagnosis. In addition, we have also discussed in other literatures that different model structures and algorithms are used for pneumonia diseases to realize the detection of pneumonia images. The literature [22] uses deep learning diagnostic tools to further prove the universal applicability of artificial intelligence in the diagnosis of pneumonia in children with chest X-ray. In the literature [37], using an improved version of CNN technology: feature extractor and a classifier (s-type activation function), [49] proposed multi-core deconvolution (MD-Conv), which has greatly improved the recall rate. In the literature [47], comparing Xception with the Vgg16 model, the test results show that the accuracy of the Vgg16 network exceeds that of Xception. However, Xception has achieved more successful results in detecting pneumonia cases. This experiment proposes a further improvement based on Xception combined with the LSTM algorithm, which is based on the model structure of ordinary Xception.

This study also explored the achievements in tuberculosis disease [50] under the same model technology. Our experimental research on medical image recognition and classification shows that the accuracy, recall, precision, and F1 of chest X-ray images of pneumonia are 0.96, 0.91, 0.98, and 0.94, respectively. At the same time, in this experiment, we applied this method to the data set of patients with tuberculosis disease, and obtained accuracy, recall, precision, and F1 of 0.99, 0.99, 1, 0.99 through model testing. It further provides a more transparent and recognizable diagnosis.

## Summary and discussion

This experiment designed a new network model in order to better realize the classification of children’s chest X-ray images and improve the accuracy between categories. At the same time, it is also better to make a modest effort in the research of medical image classification. In the experiment, we mainly used the Xception network model to initialize the image information, and changed the conventional convolution to directly extract the spatial information and channel information through a convolution kernel. At the same time, based on the lightweight neural network, combined with a variant form of the cyclic neural network, LSTM, in addition to extracting the basic image feature sequence, the sequence time information can be better mined. Because image acquisition consumes a lot of manpower and material resources, acquiring extensive training data is a difficult task. In this experiment, this problem is solved by using transfer learning and data enhancement techniques, and it also avoids overfitting to add penalty weights to the cost hyperparameters, and uses the square root of the sum of squares of the historical gradients controlled by the attenuation coefficient of the RMSprop optimizer, so that each The learning rate of the parameters is the most suitable. The traditional cross-entropy loss can not effectively solve the correlation of the training samples, combined with the chi-square test, fusion of the correlation between the classification variables between the two loss functions. Our method can outperform the benchmark model and some of the latest work results. On the same benchmark, the accuracy of this experiment reaches 0.96.

In future research work, we will further strengthen data preprocessing, improve model generalization capabilities, and continue to optimize our methods so that our research can be applied to a wider range of medical technologies.
